# Supplementary material for: CoCUT&Tag maps linked chromatin states at single-molecule, single-cell resolution
Source: bioRxiv. 2026 Apr 29:2026.04.27.721191. Preprint. [Version 1] doi: 10.64898/2026.04.27.721191 (PMC13142410; doi:10.64898/2026.04.27.721191)
Supplement: 3 [file NIHPP2026.04.27.721191v1-supplement-3.pdf]

bioRxiv preprint doi: https://doi.org/10.64898/2026.04.27.721191; this version posted April 29, 2026. The copyright holder for this preprint (which was not certified by peer review) is the author/funder, who has granted bioRxiv a license to display the preprint in perpetuity. It is made available under aCC-BY 4.0 International license.

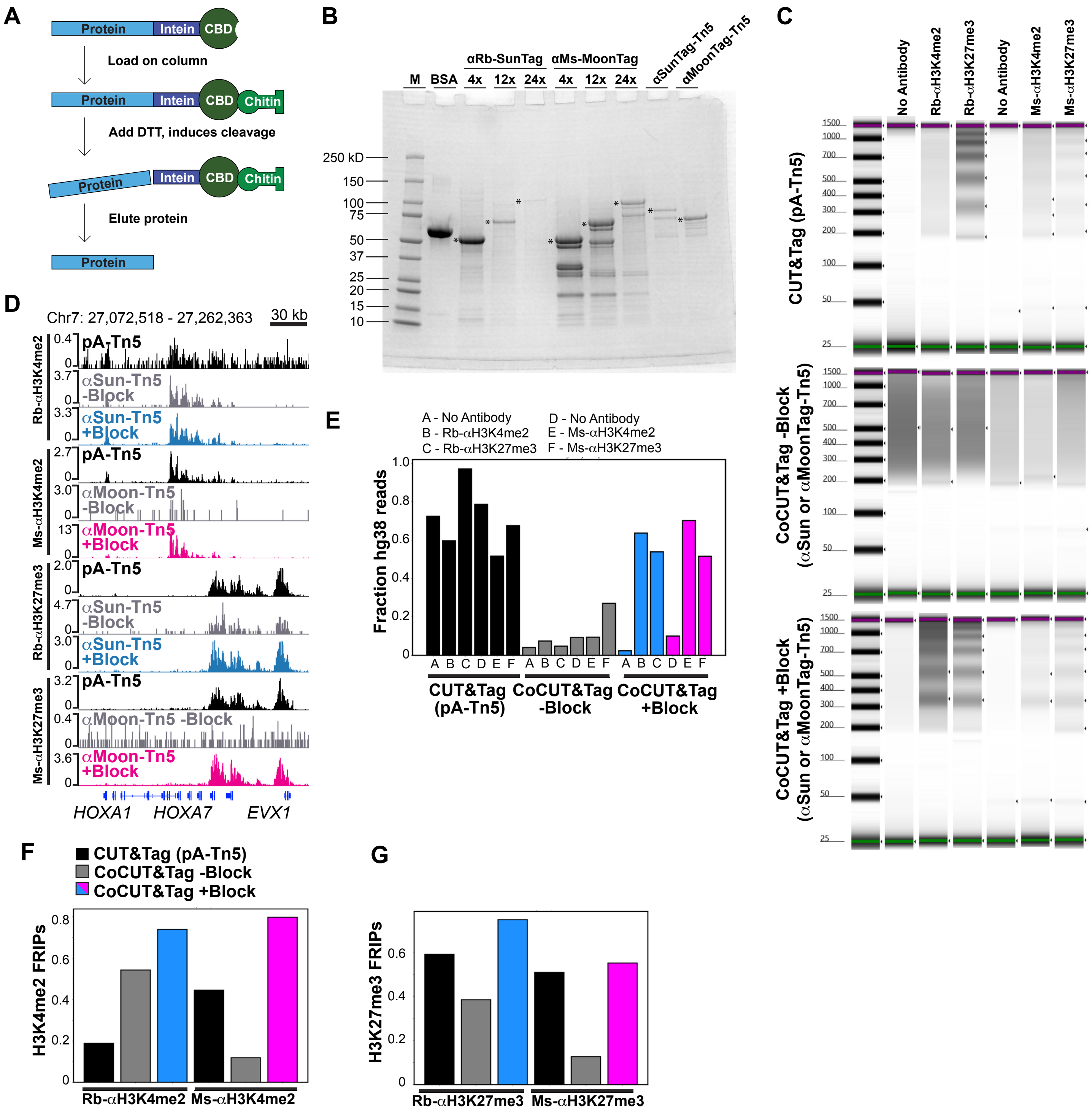


**Supplementary Figure 1. CoCUT&Tag reagent development and optimization for bulk profiling.** **(A)** All synthetic proteins in the CoCUT&Tag toolkit were expressed in E.coli fused to an N-terminal intein and chitin-binding domain (CBD), enabling purification by binding to chitin resin followed by DTT induced reduction and cleavage of the intein. **(B)** SDS-Page gel stained with Coomassie showing the 4x, 12x, and 24x anti-rabbit-SunTag and anti-rabbit-MoonTag antigen-peptide repeats and the anti-SunTag-Tn5 and anti-MoonTag-Tn5 fusion proteins. Protein bands of the expected size are indicated by an asterisk. **(C)** Tape station electrophoresis gels showing the fragment-size distributions of Standard CUT&Tag (top) and bulk CoCUT&Tag libraries generated in the absence of blocking agents (-Block; middle) or with 0.5% (w/v) BSA and Casein added to the Wash Buffers (+Block; bottom). The addition of blocking agents restores the nucleosomal laddering pattern in the CoCUT&Tag libraries. **(D)** Genome browser tracks of the *HOXA* locus in RS4;11 cells showing improved recovery of on-target chromatin signal after addition of block. **(E)** Fraction of sequenced reads aligning to the human genome for standard CUT&Tag and CoCUT&Tag libraries generated with or without block. Blocking improves the mapping rate. **(F)** Fraction of reads in peaks (FRiP) for H3K4me2 in blocked and unblocked CoCUT&Tag libraries compared with standard CUT&Tag. After blocking, CoCUT&Tag achieves FRiP values comparable to or better than standard CUT&Tag. **(G)** Same as (F) but showing the FRIPs for H3K27me3.

bioRxiv preprint doi: https://doi.org/10.64898/2026.04.27.721191; this version posted April 29, 2026. The copyright holder for this preprint (which was not certified by peer review) is the author/funder, who has granted bioRxiv a license to display the preprint in perpetuity. It is made available under aCC-BY 4.0 International license.

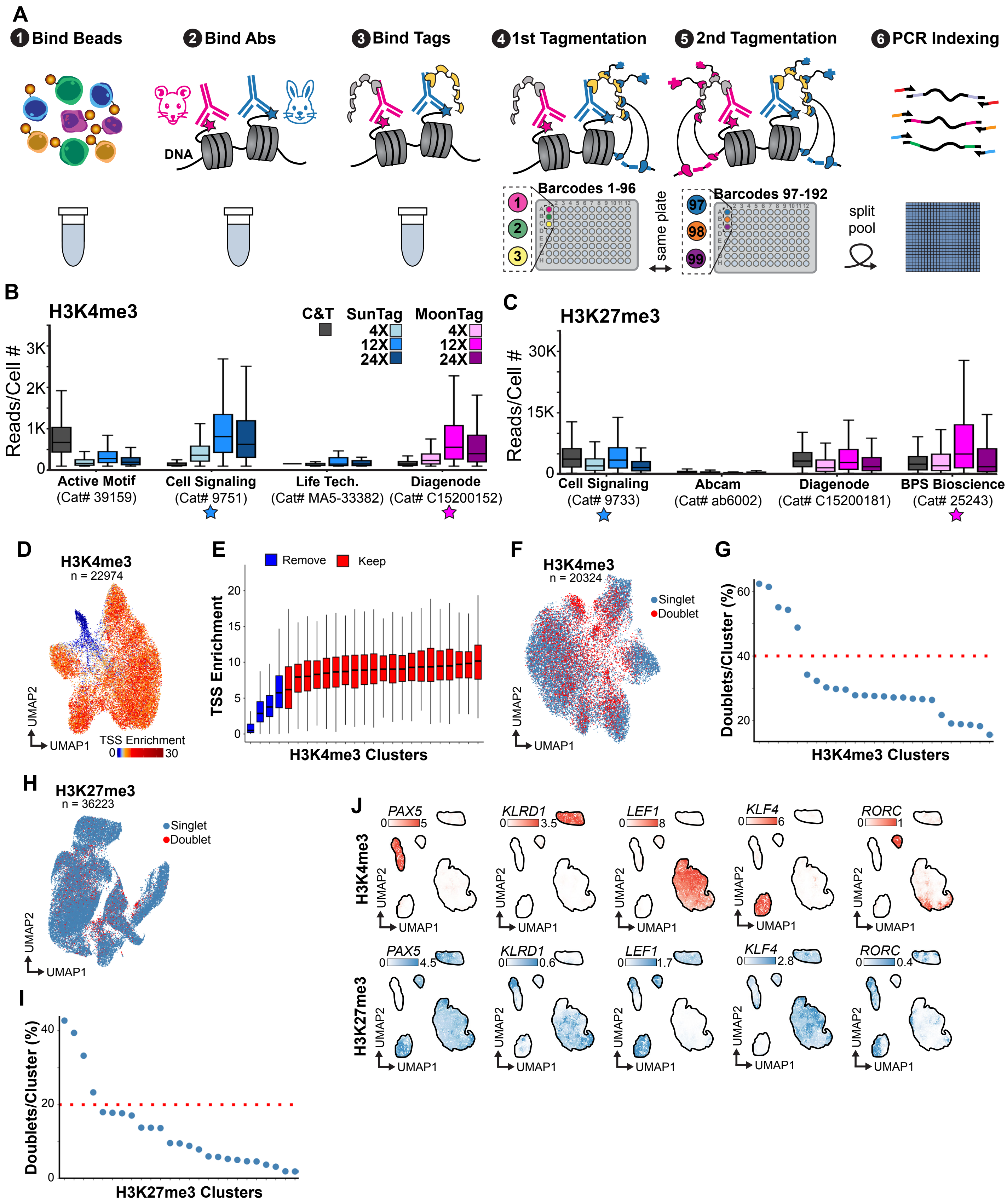


**Supplementary Figure 2. Single-cell CoCUT&Tag workflow, antibody optimization, and PBMC quality control. (A)** Combinatorial indexing workflow for single-cell CoCUT&Tag. (1) Whole cells were permeabilized, bound to magnetic beads, and lightly crosslinked to prevent aggregation, after which (2) rabbit and mouse primary antibodies were bound in bulk, (3) followed by binding of the corresponding SunTag and MoonTag scaffolds. (4) Cells were then distributed across a 96-well plate for sequential rounds of anti-SunTag-Tn5 binding and tagmentation, (5) followed by anti-MoonTag-Tn5 binding and tagmentation. Keeping cells arrayed in the same plate preserves the cellular linkage between the barcodes from the first and second round of tagmentation. (6) This is fol-

bioRxiv preprint doi: https://doi.org/10.64898/2026.04.27.721191; this version posted April 29, 2026. The copyright holder for this preprint (which was not certified by peer review) is the author/funder, who has granted bioRxiv a license to display the preprint in perpetuity. It is made available under aCC-BY 4.0 International license.

lowed by split-pool indexing during PCR on a nanowell dispenser. This approach yields up to 50,000 single-cell profiles per reaction, and starting with 1 million cells typically yields enough material for 4 split-pooling reactions. **(B,C)** Antibody and scaffold optimization for H3K4me3 (B) and H3K27me3 (C). Selected rabbit and mouse antibodies perform comparably to or better than standard CUT&Tag when paired with 12x SunTag or MoonTag constructs. **(D,E)** PBMC H3K4me3 quality control. UMAP projection colored by TSS enrichment (D) and cluster-level TSS enrichment used to identify low-quality clusters for removal (E). **(F,G)** Doublet detection and removal for PBMC H3K4me3 data using ArchR-based synthetic doublet scoring and cluster-level doublet enrichment. **(H,I)** Analogous doublet filtering and quality control for PBMC H3K27me3 data. **(J)** PBMC weighted nearest neighbor UMAP-embedding colored by the ArchR-imputed H3K4me3 gene scores (top) and H3K27me3 gene scores (bottom), for marker genes enriched in B-cells (*PAX5*), NK Cells (*KLRD1*), T cells (*LEF1*), monocytes (*KLF4*) and Cytotoxic T cells (*RORC*).

bioRxiv preprint doi: https://doi.org/10.64898/2026.04.27.721191; this version posted April 29, 2026. The copyright holder for this preprint (which was not certified by peer review) is the author/funder, who has granted bioRxiv a license to display the preprint in perpetuity. It is made available under aCC-BY 4.0 International license.

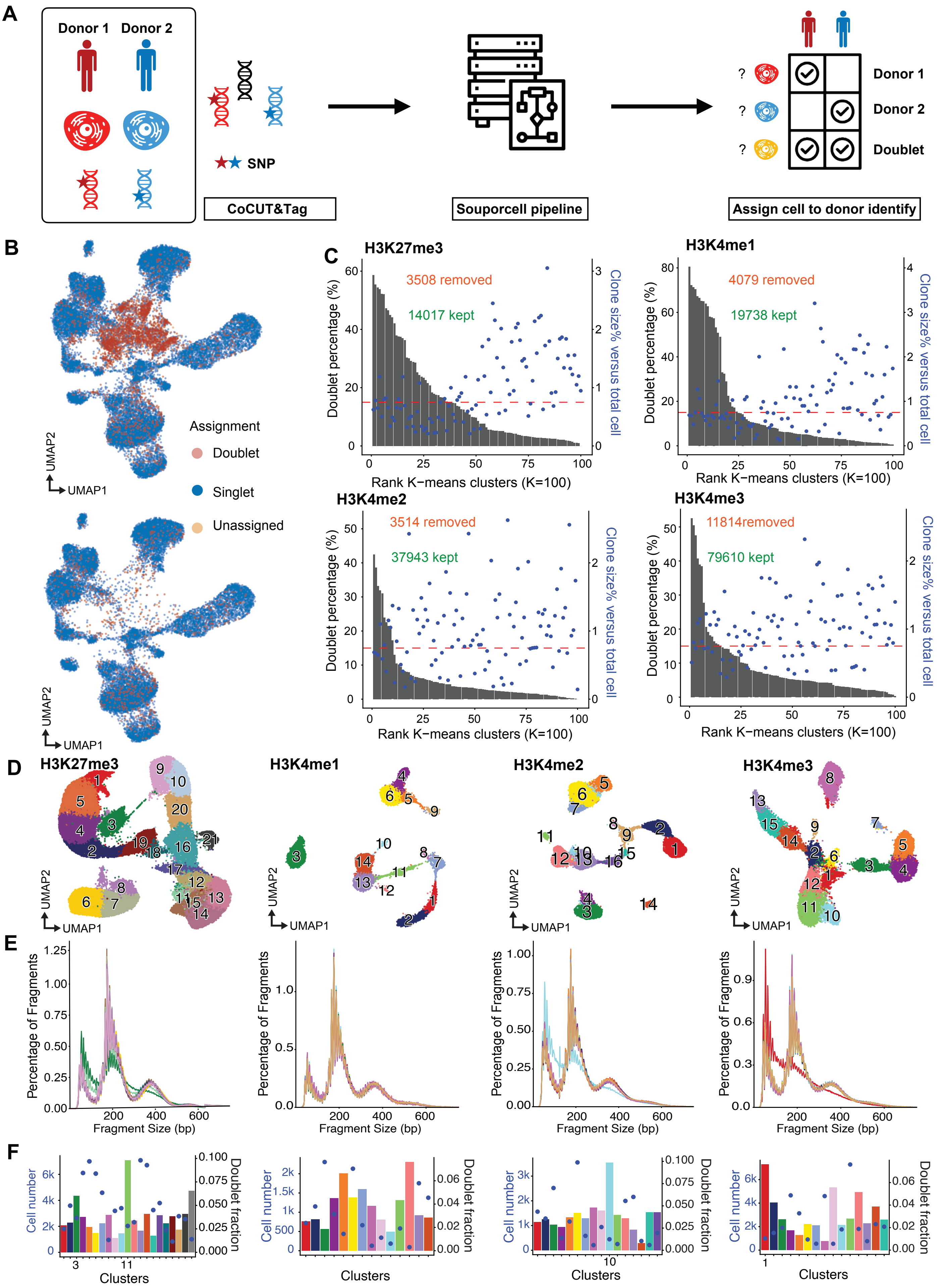

bioRxiv preprint doi: https://doi.org/10.64898/2026.04.27.721191; this version posted April 29, 2026. The copyright holder for this preprint (which was not certified by peer review) is the author/funder, who has granted bioRxiv a license to display the preprint in perpetuity. It is made available under aCC-BY 4.0 International license.

**Supplementary Figure 3. Mixed-donor doublet detection and quality control for bone marrow CoCUT&Tag. (A)** Experimental and computational workflow for mixed-donor doublet detection. Bone marrow cells from multiple donors were pooled and analyzed using SNP-based donor assignment to identify singlets and doublets. **(B)** UMAP embeddings colored by donor-assigned singlets, doublets, and unassigned cells before and after the first round of cluster-based removal of doublet-enriched populations. **(C)** Quantification of doublet enrichment across K-means clusters for H3K27me3, H3K4me1, H3K4me2, and H3K4me3 datasets, used to identify clusters removed in the first round of filtering. **(D)** Reclustering and embedding after initial doublet-enriched cluster removal. **(E)** Fragment-size distributions of clusters from (D). Residual low-quality clusters show weakened nucleosomal laddering, consistent with degraded chromatin or poor cellular integrity. **(F)** Residual low-quality clusters also retain elevated doublet fractions and were removed in a second round of filtering.

bioRxiv preprint doi: https://doi.org/10.64898/2026.04.27.721191; this version posted April 29, 2026. The copyright holder for this preprint (which was not certified by peer review) is the author/funder, who has granted bioRxiv a license to display the preprint in perpetuity. It is made available under aCC-BY 4.0 International license.

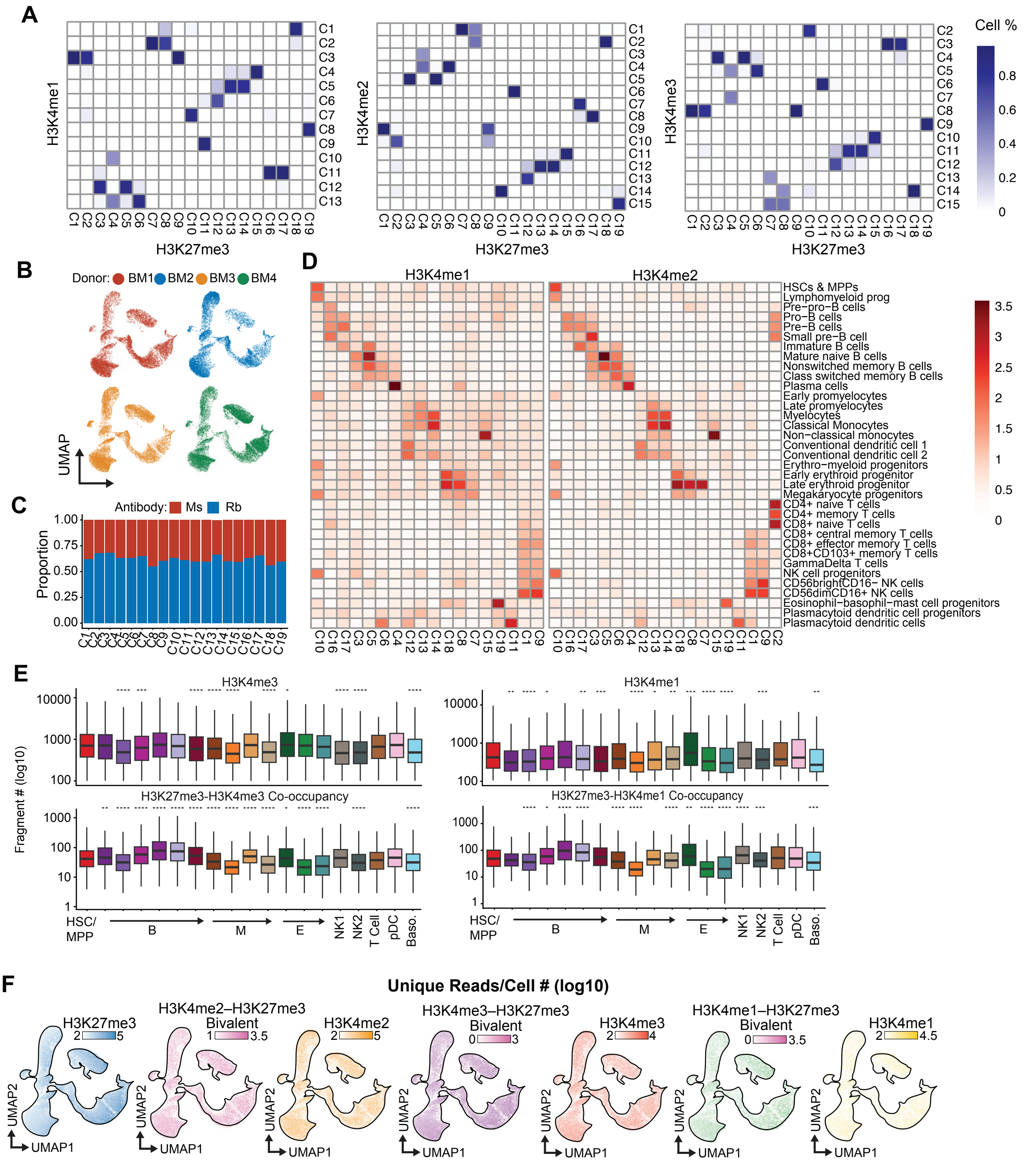


**Supplementary Figure 4. Additional validation of filtered bone marrow CoCUT&Tag datasets. (A)** Correspondence of cluster identities across paired H3K27me3 and H3K4me1, H3K4me2 and H3K4me3 datasets. Cells from a single cluster in the H3K4 methylation embeddings are often split into multiple clusters by the paired H3K27me3 profiles, reinforcing the added power of H3K27me3 profiles for resolving hematopoietic cell states. **(B)** Distribution of cells from each donor across the H3K27me3 embedding, showing broad donor mixing across hematopoietic populations. **(C)** Distribution of cells profiled with rabbit and mouse H3K27me3 antibodies across clusters, indicating minimal antibody-specific batch effects. **(D)** Heatmaps of cluster-specific ArchR-imputed genes from the H3K4me1 and H3K4me2 datasets alongside expression of the same genes in a reference single-cell RNA-seq dataset with surface marker-defined hematopoietic identities. **(E)** Reads per cell for H3K27me3-H3K4me1 co-occupancy, H3K27me3-H3K4me3 co-occupancy, H3K4me1, and H3K4me3 across retained bone marrow cell states. **(F)** H3K27me3 BMMC UMAP embedding, colored by the unique read counts for each mark.

bioRxiv preprint doi: https://doi.org/10.64898/2026.04.27.721191; this version posted April 29, 2026. The copyright holder for this preprint (which was not certified by peer review) is the author/funder, who has granted bioRxiv a license to display the preprint in perpetuity. It is made available under aCC-BY 4.0 International license.

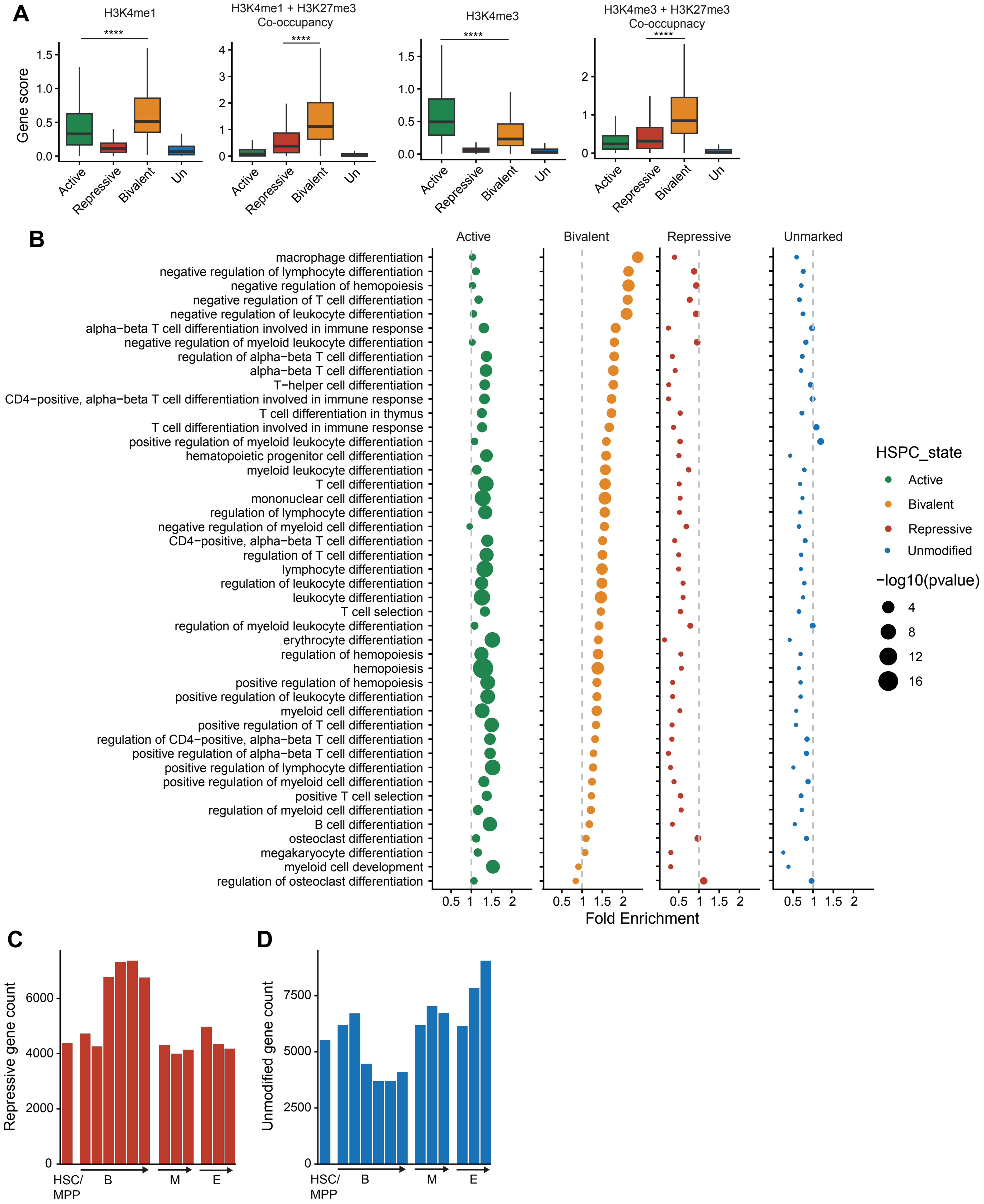


**Supplementary Figure 5. Additional analysis of bivalent, repressed, and unmarked gene states across hematopoiesis. (A)** Boxplots showing the H3K4me1, H3K4me3 and corresponding H3K27me3 co-occupancy gene scores for genes classified as Active, Repressive, Bivalent and Unmarked. **(B)** Gene Ontology enrichment across active, bivalent, repressed, and unmarked gene classes in HSC/MPPs. Bivalent genes are enriched for hematopoietic developmental programs. **(C)** Absolute number of repressed genes across the HSC/MPP and lineage-committed B, M, and E clusters. **(D)** Absolute number of unmarked genes across the HSC/MPP and lineage-committed B, M, and E clusters.

bioRxiv preprint doi: https://doi.org/10.64898/2026.04.27.721191; this version posted April 29, 2026. The copyright holder for this preprint (which was not certified by peer review) is the author/funder, who has granted bioRxiv a license to display the preprint in perpetuity. It is made available under aCC-BY 4.0 International license.

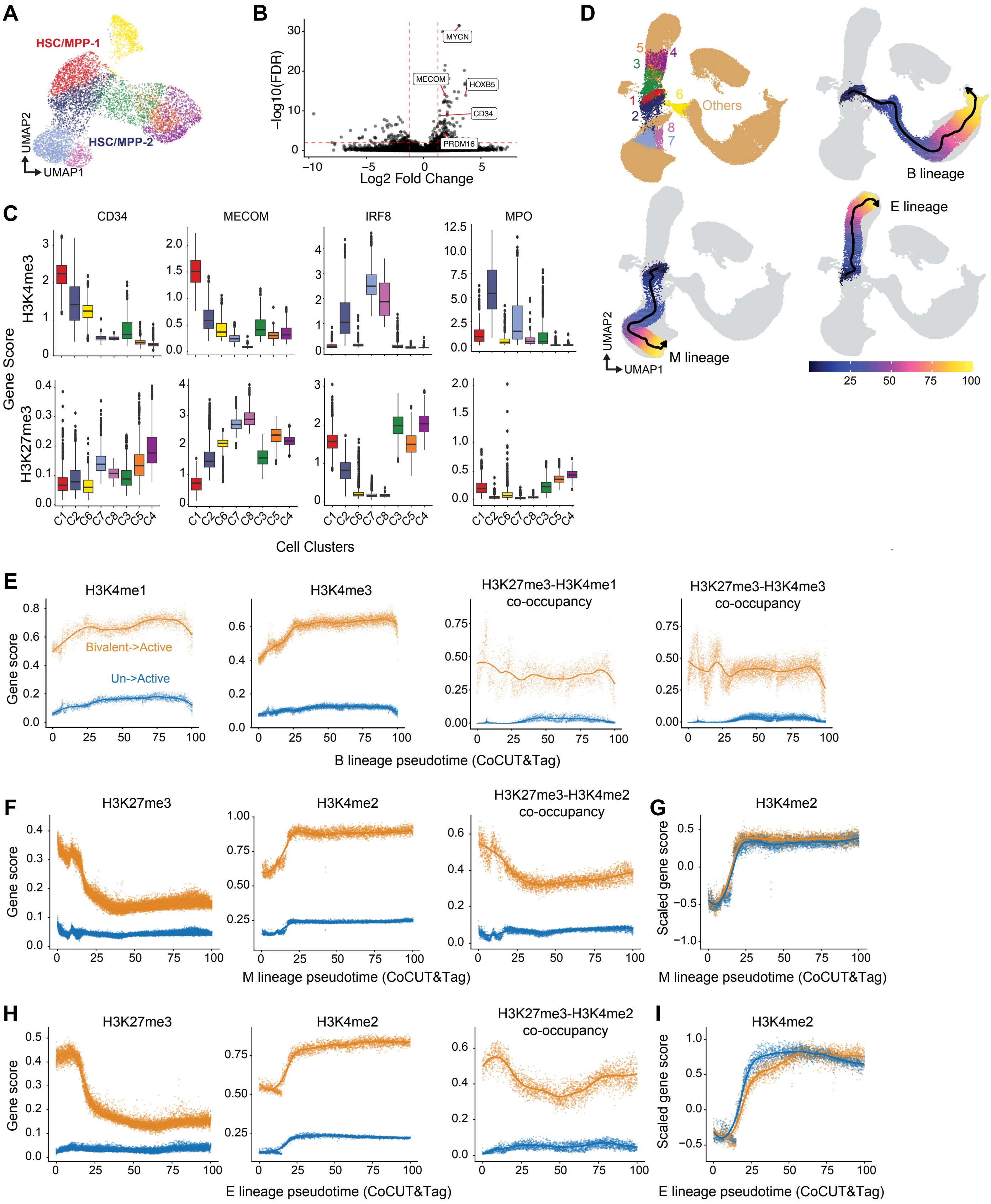


**Supplementary Figure 6. Definition of the HSC/MPP root state and additional pseudotime analyses. (A)** Refined clustering of the HSC/MPP compartment used to define the root population for developmental trajectory analysis. **(B)** Differentiall analysis of the H3K4me3 gene scores showing the HSC genes *MYCN, MECOM*, *HOXB5, PRDM16* and *CD34* were enriched for H3K4me3 in the HSC/MPP-1 cluster from (A), and this cluster was defined as the root for trajectory analysis. **(C)** Distribution of representative HSC-associated genes (*MECOM*, *CD34*) and myeloid-associated genes (*IRF8*, *MPO*) across the refined HSC/MPP subclusters using

bioRxiv preprint doi: https://doi.org/10.64898/2026.04.27.721191; this version posted April 29, 2026. The copyright holder for this preprint (which was not certified by peer review) is the author/funder, who has granted bioRxiv a license to display the preprint in perpetuity. It is made available under aCC-BY 4.0 International license.

H3K4me3 and H3K27me3 ArchR-imputed gene scores. **(D)** B, M, and E lineage pseudotime trajectories overlaid on the integrated hematopoietic embedding. **(E)** Smoothed CoCUT&Tag gene scores for H3K4me1, H3K4me3, H3K27me3-H3K4me1 co-occupancy, and H3K27me3-H3K4me3 co-occupancy across B-lineage pseudotime for genes that transition from bivalent-to-active chromatin or from unmarked-to-active chromatin. **(F)** Same as (E) but showing gene scores across the myeloid-lineage trajectory. **(G)** Scaling the H3K4me2 gene scores from (F) shows that unmarked and bivalent genes gain H3K4me2 with similar timing. **(H)** Same as (E) but showing gene scores across the erythroid-lineage trajectory. **(I)** Same as (G) but showing the scaled H3K4me2 gene scores from the erythroid lineage trajectory.

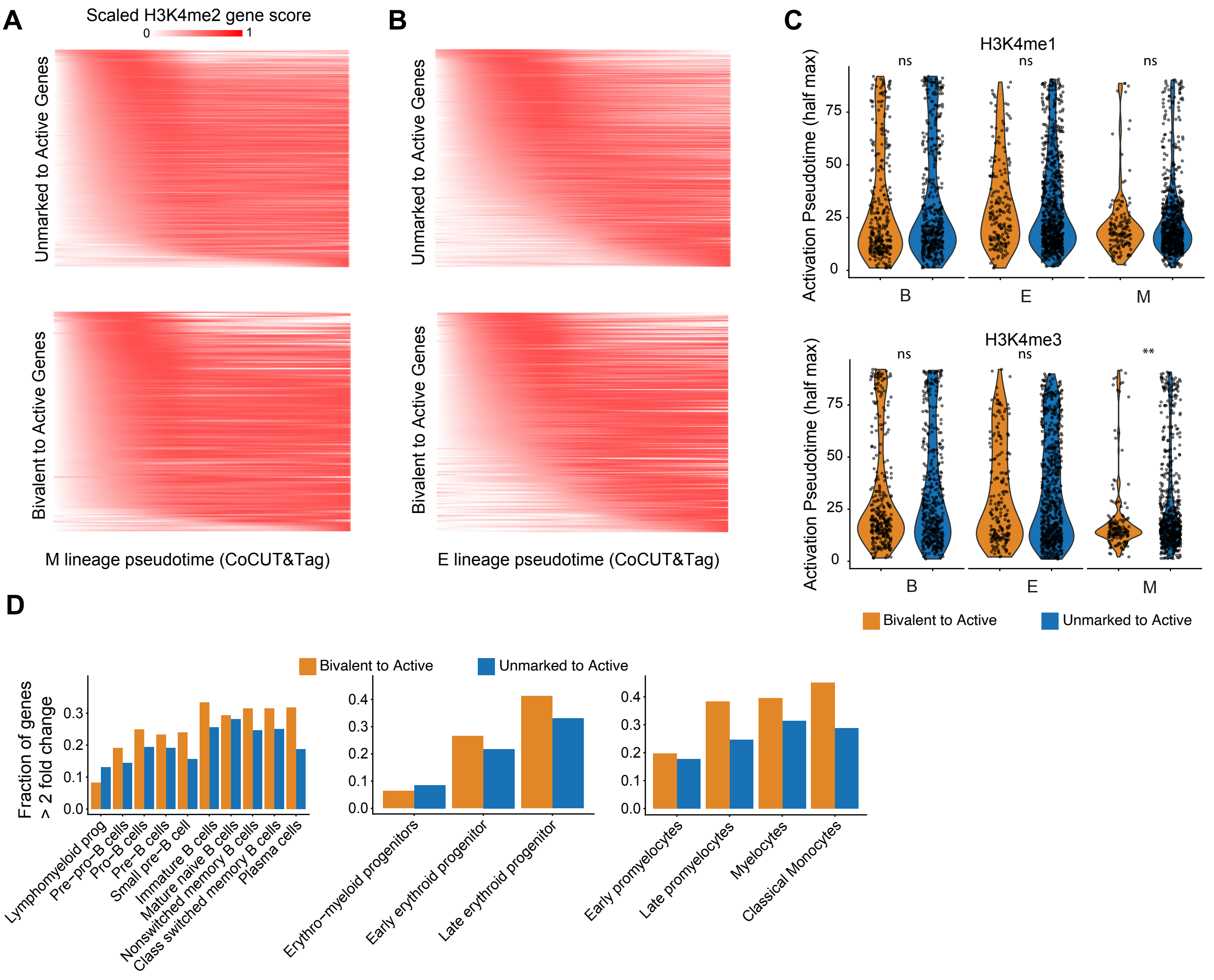


**Supplementary Figure 7. Additional comparisons of gene activation timing and magnitude. (A)** Heatmap comparing the H3K4me2 gene scores across myeloid-lineage pseudotime for the unmarked genes (top) versus the bivalent genes (bottom) that transitioned to active chromatin. Genes are rank ordered according to the point they reach the half max H3K4me2 gene scores in pseudotime. **(B)** Same as (A) but for the erythroid lineage. **(C)** Violin plot showing the H3K4me1 (top) and H3K4me3 (bottom) activation timing (half-max point in pseudotime) is similar for bivalent and unmarked genes. **(D)** Proportion of genes with greater than two-fold induction across pseudotime in the B, E, and M lineages for genes that transition from bivalent-to-active chromatin or from unmarked-to-active chromatin.

bioRxiv preprint doi: https://doi.org/10.64898/2026.04.27.721191; this version posted April 29, 2026. The copyright holder for this preprint (which was not certified by peer review) is the author/funder, who has granted bioRxiv a license to display the preprint in perpetuity. It is made available under aCC-BY 4.0 International license.

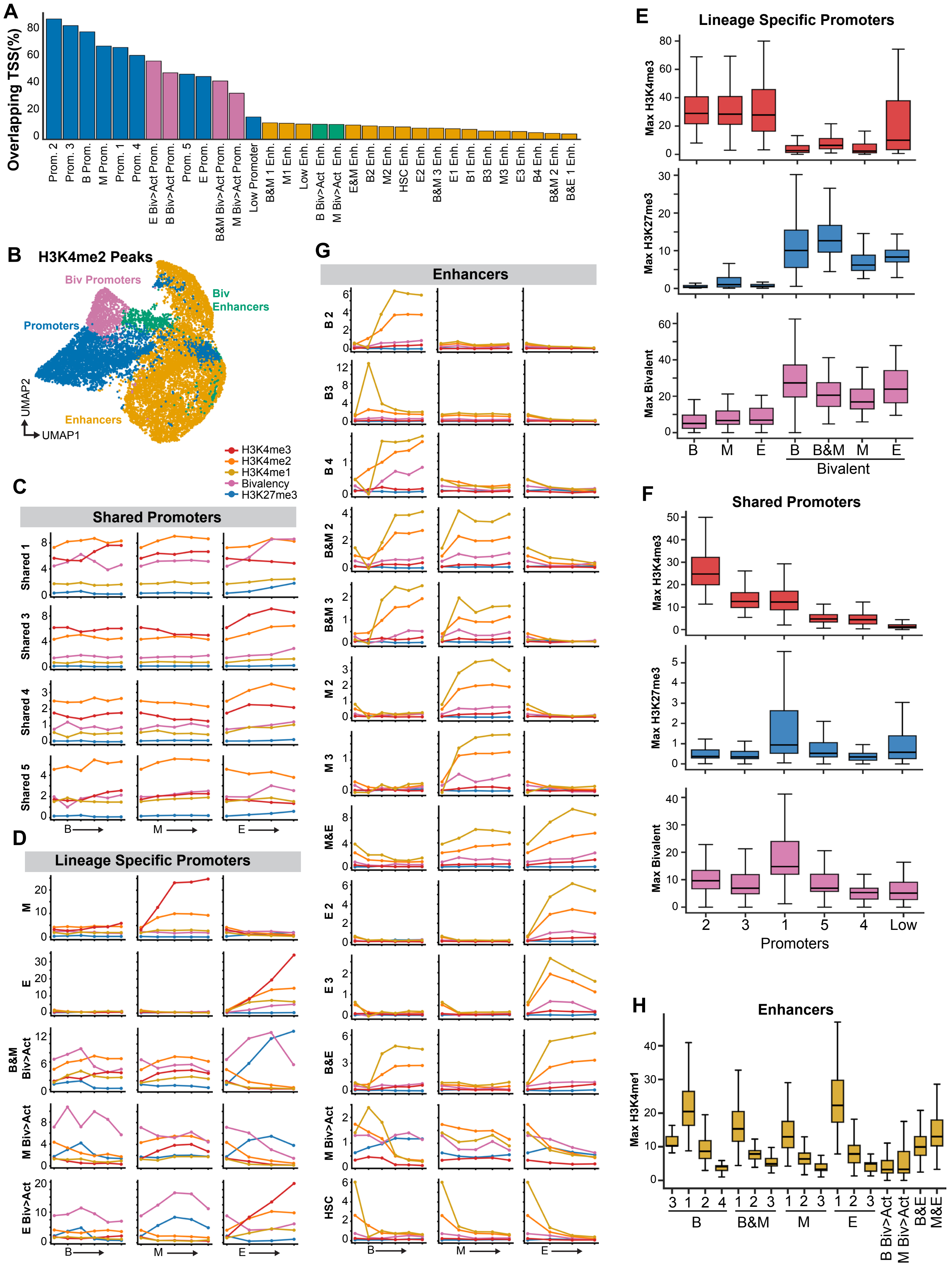

bioRxiv preprint doi: https://doi.org/10.64898/2026.04.27.721191; this version posted April 29, 2026. The copyright holder for this preprint (which was not certified by peer review) is the author/funder, who has granted bioRxiv a license to display the preprint in perpetuity. It is made available under aCC-BY 4.0 International license.

**Supplementary Figure 8. Additional promoter and enhancer subclass definitions.**
**(A,B)** Additional promoter and enhancer subclass annotations. Promoter classes are strongly enriched near annotated transcription start sites, whereas enhancer classes are predominantly distal. **(C,D)** Average chromatin profiles for general and lineage-specific promoter subclasses across the B, M, and E lineages. Lineage-specific promoters show the highest H3K4me3, whereas bivalent promoters retain elevated H3K27me3 and bivalent signal. **(E,F)** Quantification of H3K4me3, H3K27me3, and bivalent signal across lineage-specific promoter classes (E) and general promoter classes (F). **(G)** Additional putative enhancer subclasses, including lineage-specific enhancer groups with the strongest H3K4me1 signal and rare bivalent enhancer classes. **(H)** Quantification of maximal H3K4me1 signal across enhancer subclasses, showing that the strongest lineage-specific enhancer groups tend to have the highest H3K4me1 levels.

bioRxiv preprint doi: https://doi.org/10.64898/2026.04.27.721191; this version posted April 29, 2026. The copyright holder for this preprint (which was not certified by peer review) is the author/funder, who has granted bioRxiv a license to display the preprint in perpetuity. It is made available under aCC-BY 4.0 International license.

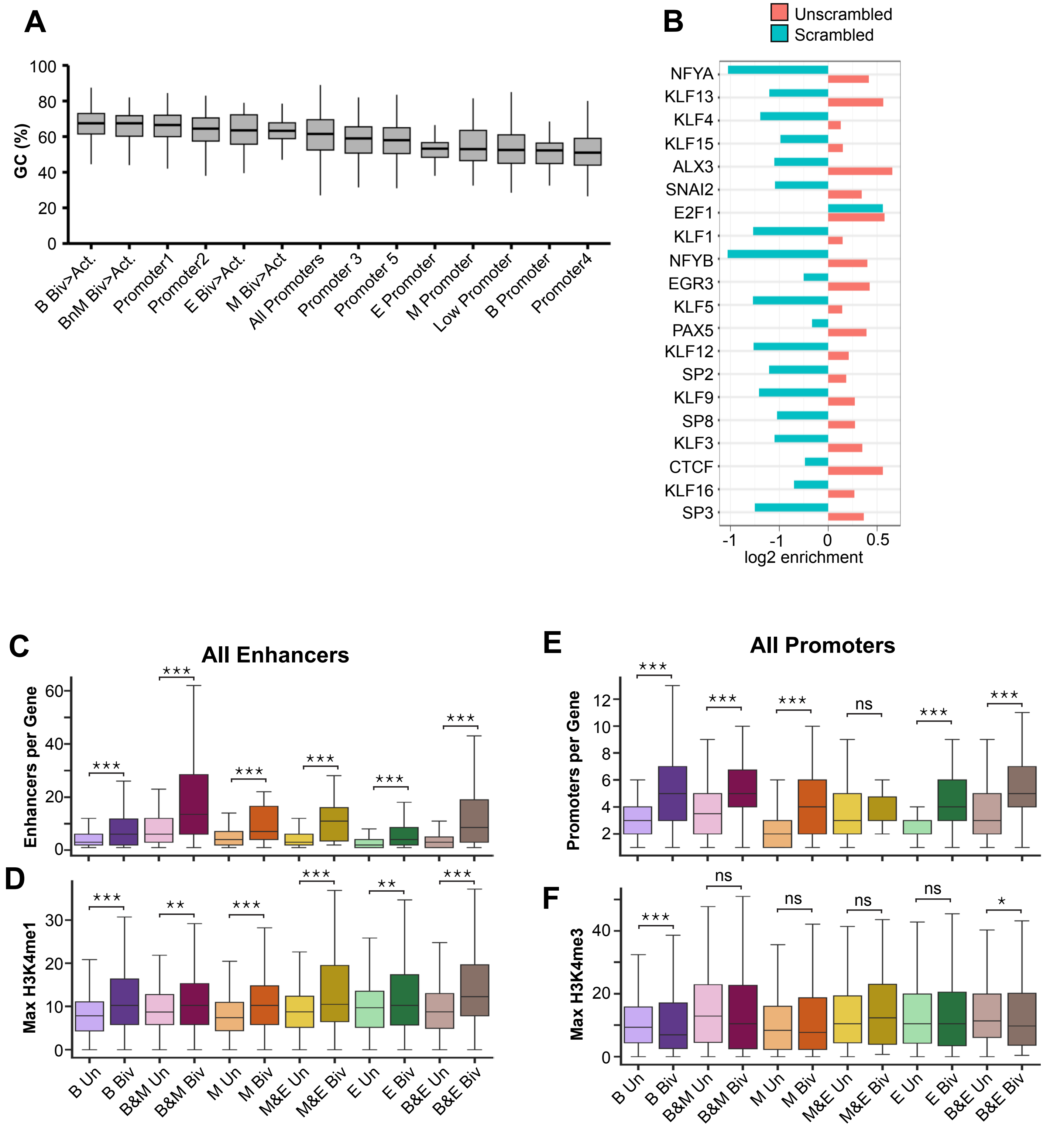


**Supplementary Figure 9. Additional promoter and enhancer features distinguishing bivalent-to-active and unmarked-to-active genes.** **(A)** GC content across promoter classes. **(B)** Numerous motifs enriched in the Shared Promoter 1 subclass are GC-rich, including SP/KLF-family factors, CTCF, and PAX5. However, these motifs are no longer enriched after sequence scrambling, indicating their enrichment cannot be explained by GC% alone. **(C,D)** Quantification of enhancer number per gene (C) and enhancer H3K4me1 signal (D) using all enhancer classes rather than only the top lineage-associated enhancer groups. Bivalent-to-active genes retain more enhancers with stronger H3K4me1 signal than unmarked-to-active genes under this broader analysis as well. **(E,F)** Promoter abundance per gene (E) and promoter H3K4me3 signal (F) across gene transition classes. Bivalent-to-active genes tend to reside in more promoter-rich domains than unmarked-to-active genes, but promoters near the bivalent genes are not marked by higher H3K4me3 signal.

bioRxiv preprint doi: https://doi.org/10.64898/2026.04.27.721191; this version posted April 29, 2026. The copyright holder for this preprint (which was not certified by peer review) is the author/funder, who has granted bioRxiv a license to display the preprint in perpetuity. It is made available under aCC-BY 4.0 International license.

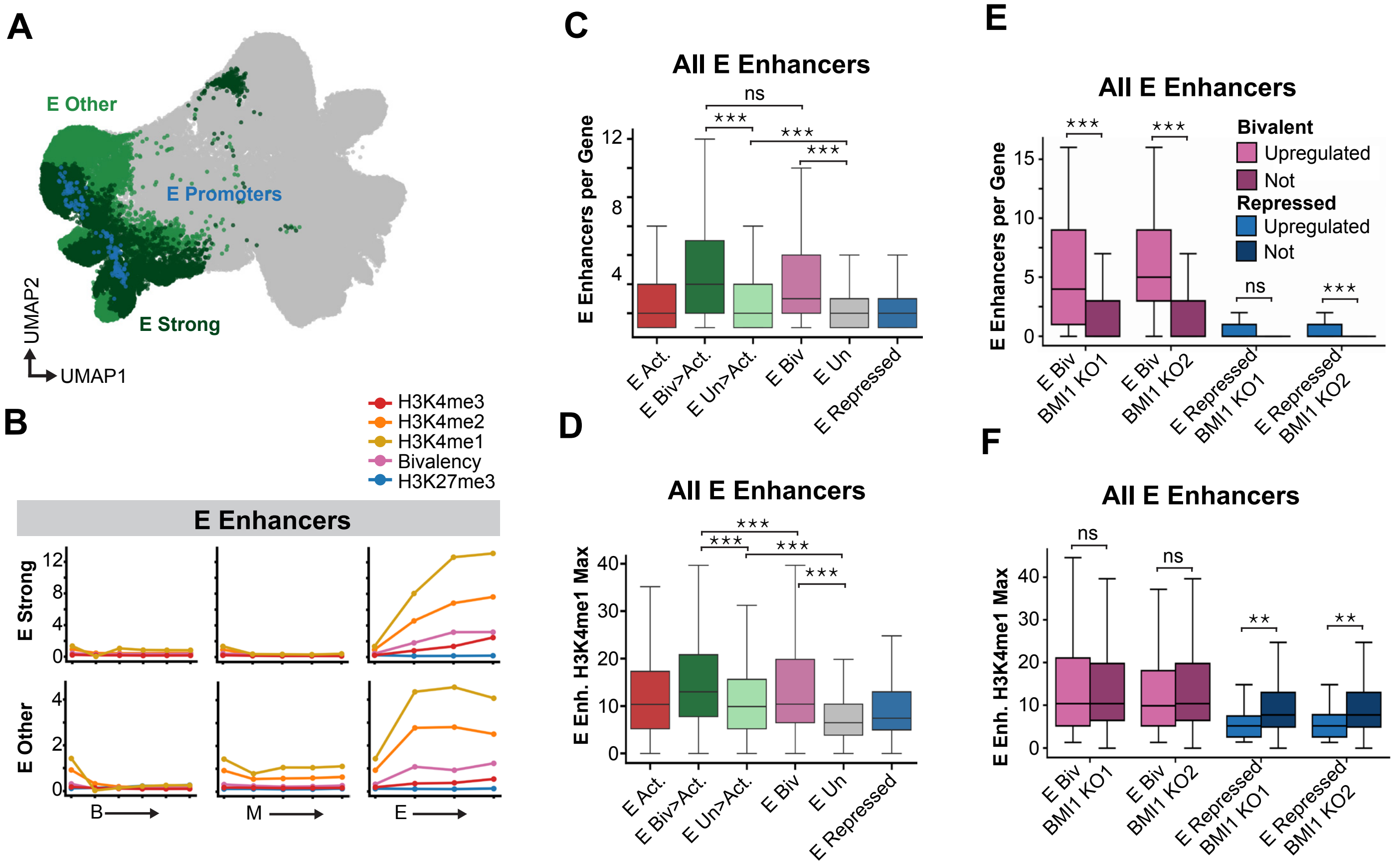


**Supplementary Figure 10. Robustness of erythroid enhancer analyses across enhancer definitions. (A)** UMAP of all H3K4me3 peaks with strong erythroid enhancers, other erythroid enhancers, and erythroid promoter elements indicated. **(B)** Average chromatin profiles for strong and other erythroid enhancer classes across the B, M, and E lineages. **(C,D)** Number of erythroid enhancers per gene (C) and maximal erythroid enhancer H3K4me1 signal (D) calculated using all erythroid enhancer classes rather than only the strongest enhancer groups. Genes transitioning from bivalent-to-active chromatin retain more erythroid enhancers with stronger H3K4me1 signal than genes remaining bivalent, remaining unmarked, or transitioning from unmarked-to-active chromatin. **(E,F)** *BMI1* knockout analysis repeated using all erythroid enhancer classes. Among genes classified as bivalent in throughout the erythroid lineage, those upregulated after *BMI1* loss carry more erythroid enhancers per gene than non-upregulated bivalent genes, whereas repressed genes do not show the same pattern. Maximal enhancer H3K4me1 signal shows weaker separation than enhancer number.
